# Finite Element Modeling and Performance Evaluation of Piezoelectric Energy Harvesters with Various Piezoelectric Unit Distributions

**DOI:** 10.3390/ma14061405

**Published:** 2021-03-14

**Authors:** Cong Du, Pengfei Liu, Hailu Yang, Gengfu Jiang, Linbing Wang, Markus Oeser

**Affiliations:** 1 Institute of Highway Engineering (ISAC), RWTH Aachen University, 52074 Aachen, Germany; du@isac.rwth-aachen.de (C.D.); gengfu.jiang@rwth-aachen.de (G.J.); oeser@isac.rwth-aachen.de (M.O.); 2 National Center for Materials Service Safety, University of Science and Technology Beijing (USTB), Beijing 100083, China; 3 Research and Development Center of Transport Industry of New Materials, Technologies Application for Highway Construction and Maintenance, Beijing 100088, China; 4 Joint USTB Virginia Tech Lab on Multifunctional Materials, University of Science and Technology Beijing (USTB), Beijing 100083, China; wangl@vt.edu

**Keywords:** piezoelectric energy harvester, finite element simulation, piezoelectric unit distributions, electrical potential and energy, von Mises stress

## Abstract

The piezoelectric energy harvester (PEH) is a device for recycling wasted mechanical energy from pavements. To evaluate energy collecting efficiency of PEHs with various piezoelectric unit distributions, finite element (FE) models of the PEHs were developed in this study. The PEH was a square of 30 cm × 30 cm with 7 cm in thickness, which was designed according to the contact area between tire and pavement. Within the PEHs, piezoelectric ceramics (PZT-5H) were used as the core piezoelectric units in the PEHs. A total of three distributions of the piezoelectric units were considered, which were 3 × 3, 3 × 4, and 4 × 4, respectively. For each distribution, two diameters of the piezoelectric units were considered to investigate the influence of the cross section area. The electrical potential, total electrical energy and maximum von Mises stress were compared based on the computational results. Due to the non-uniformity of the stress distribution in PEHs, more electrical energy can be generated by more distributions and smaller diameters of the piezoelectric units; meanwhile, more piezoelectric unit distributions cause a higher electrical potential difference between the edge and center positions. For the same distribution, the piezoelectric units with smaller diameter produce higher electrical potential and energy, but also induce higher stress concentration in the piezoelectric units near the edge.

## 1. Introduction

With the development of economy and society, the number of traffic loads on asphalt pavements increases in the recent years. During the service life of pavements, millions of axle’s loads causes large amounts of wasted mechanical energy. As a remedy, new technologies have been developed and applied to recycle the energy from urban roads and highways by converting them to other types of energy resources. One such example is the energy harvesting technology using piezoelectric and magnetostrictive materials, which can convert the mechanical energy generated by the traffic loads to electrical energy [1,2,3,4,5,6]. Among others, piezoelectric energy harvester (PEH) shows significant advantages for maintenance of energy output along with traffic flow without being influenced by weather, environmental temperature, and so on [7].

Many researches have been conducted focusing on the piezoelectric materials for the energy harvesting [8,9]. For examples, Anton and Sodano [10] reviewed the piezoelectric materials used for energy saving, including the lead zirconate titanate, also known as piezoelectric ceramic (PZT), poly(vinylidene fluoride) (PVDF) [11], and the macro-fiber composite (MFC) [12]. They found that the PZT materials were the most commonly used piezoelectric materials in the energy harvesting due to its high efficiency. Besides, many scholars [13,14] developed the fiber-based materials in which the PZT fibers with various diameters were consisted. The results showed that a relatively small fiber-based piezoelectric power harvester can supply useable amounts of power from cyclic strain vibration in the local environment. However, the piezoelectric ceramic is very brittle, and its piezoelectric feature only works under undamaged strain conditions. In addition, the stiffness of the piezoelectric ceramic is much higher than pavement materials, which could cause the stress concentration behavior and therefore induce damages.

To address this issue, numerous researches packaged the PZT materials into PEHs using various package materials and shapes to reduce the damages and improve the energy harvesting efficiency [15]. For examples, Yesner et al. [16] developed a bridge transducer based on the cymbal design, which exhibits higher energy generation in horizontal loading condition comparing with the conventional design. Moure et al. [17] tested the electrical energy conversion of piezoelectric cymbals with 29-mm diameter, and the piezoelectric cymbal were integrated into asphalt pavements to evaluate the energy harvesting ability in normal traffic conditions. Zhao et al. [18] developed the multilayer PZT-5 stack configuration for the civil infrastructure application, and the results indicates that the analytical and numerical predications used in their research exhibited very good agreement with the experimental measurements. Xiong et al. [19] developed the PEH prototype that consists of PZT disks sealed in a protective package, and the PEHs were fabricated in the pavement to evaluate their feasibility of energy harvesting. The results showed that the energy harvesters are highly relevant to the axle configuration and magnitude of passing vehicles. Liu et al. [20,21] investigated the influence of PEHs on the structural response of asphalt pavement, which provides the basic information for improving the design of PEHs in application in pavement engineering. Zhang et al. [22] proposed a new packaging method using monomer cast nylon and epoxy resin as the main protective materials for the PEHs. The normalized output power of the PEH system was found to rely on the normalized electrical resistive load and normalized embedded depth. To further improve the efficiency of the piezoelectric energy collection, Yang et al. [23,24] developed the PEH by laboratory and in-situ tests. The PZT-5H was selected to serve as the core piezoelectric units within PEHs. Their researches successfully recycled energy from the pavements, and thus provided a useful guideline for optimization of PEH system in practical roadway applications.

The abovementioned researches provide a general overview on the piezoelectric energy harvesting on asphalt pavements. It is foreseeable that there is a high potential to harvest kinetic energy from pavement using the PEHs. However, with consideration of the laboratory cost and convenience, the design of the PEHs in current researches is still mainly based on empirical approaches, and the PEHs with higher efficiency need to be further developed.

To this end, numerical technologies, like finite element (FE) method, provide possibilities to researchers to massively and comprehensively investigate the mechanical and electrical responses of the PEHs. Zhao et al. [25] designed a cymbal for harvesting energy from asphalt pavement, and the efficiency and coupling effects with pavement of cymbals with various sizes were discussed through FE simulations. As an initial research, the FE models of the cymbals in their study were simplified to some extent, which were not directly applied in realistic pavements. Yang et al. [26] evaluated the efficiencies of PEHs in different locations in asphalt pavement based on the FE simulation, the results can be used to guide the future PEHs applications in pavement engineering. However, the PEH in Yang’s simulation was simplified as a homogeneous structure, and the details about the internal structure of the PEH were ignored.

## 2. Objectives and Outlines

**Figure 1:**
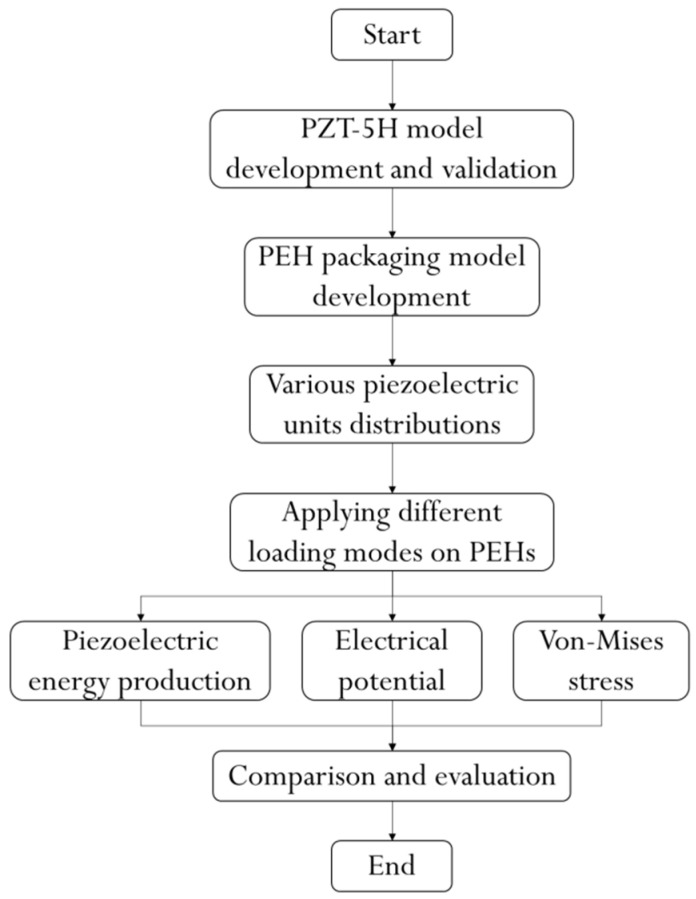
Flowchart of the study.

In this study, the piezoelectric energy harvesting efficiency of the PEH is further investigated using FE method. A flowchart as shown in Figure 1 is provided to clearly exhibit the simulation and analysis process in this study.

The internal structure of the PEH was reconstructed based on the authors’ previous research [23,24]. Various distributions of the piezoelectric units inside the PEH were modeled in FE software ABAQUS (version 2017). A total of three loading modes were applied on the PEH models to simulate the realistic non-uniform traffic loading conditions. According to the computational results, including the total electrical energy and electrical potential, the energy collecting efficiencies of the PEH were evaluated. In addition, the mechanical performances of the piezoelectric units were analyzed. At the end of this study, recommendations for the future PEH design were proposed according to the computational results.

## 3. Methodology

### 3.1. Foundation of Piezoelectric Theory

**Figure 2:**
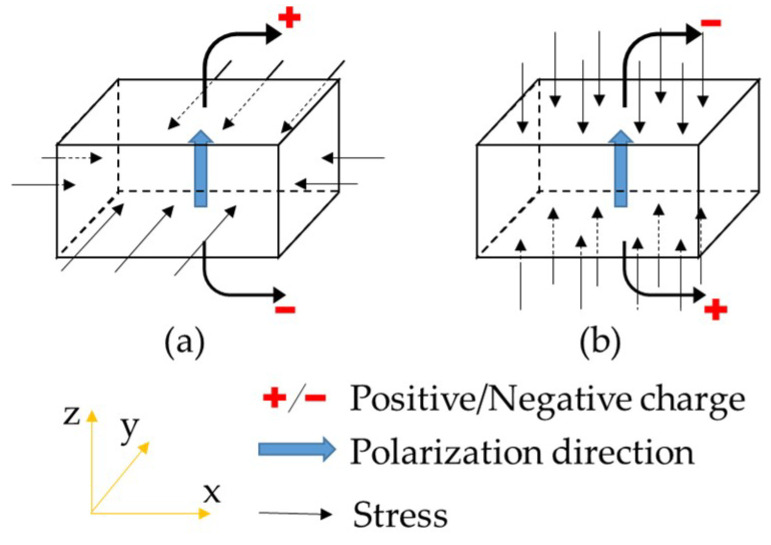
Working modes of the piezoelectric materials: (**a**) 3-1 mode; (**b**) 3-3 mode.

According to [27,28], the basic equations for the piezoelectric linear medium in this numerical study are defined as below

(1) $$\sigma_{\mathrm{ij}}{=D}_{\mathrm{ijkl}}^{E}\varepsilon_{\mathrm{kl}}\;-{\;e}_{\mathrm{mij}}^{\varphi }E_{m}$$

(2) $$q_{i}{=e}_{\mathrm{ijk}}^{\varphi }\varepsilon_{\mathrm{jk}}{\;+\;D}_{\mathrm{ij}}^{\varphi (\varepsilon )}$$

where σ*_ij_* and ε*_ij_* are stress and strain components, Pa and -, respectively; *q_i_* are the electrical flux components, V·m; *D_ijkl_* are the material stiffness, Pa; $e_{\mathrm{mij}}^{\varphi }$ are piezoelectric constant, C/m^2^; $D_{\mathrm{ij}}^{\varphi }$ are the dielectric constants, C/(V·m); *E_m_* is the electrical fields, V/m. In the above equations, the superscripts *E* and ε above a particular property indicate that the property is defined at zero electrical gradient and at zero strain, respectively. For the piezoelectric effects, two working modes are defined for the piezoelectric materials, which, respectively, are 3-1 mode and 3-3 mode [26], as shown in Figure 2.

The 3-1 mode refers to that the stress components are perpendicular to the polarization direction of the piezoelectric materials. The 3-3 stands for that the stress component is parallel to the polarization direction.

### 3.2. Prototype of the PEH

**Figure 3:**
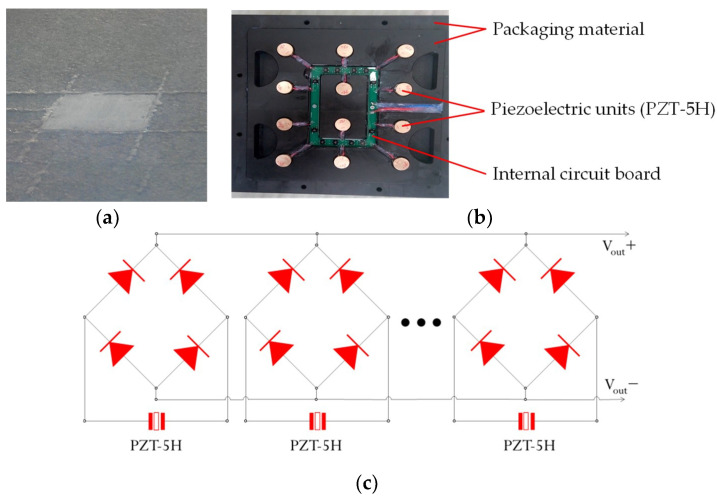
Prototype of the piezoelectric energy harvester (PEH) [23,24]: (**a**) PEH installation in pavement; (**b**) Details of PEH inside design; (**c**) Connection of the piezoelectric units.

The structure of the PEH used in this study is based on the authors’ previous researches [23,24,26]. The PEH was designed as a box and was buried in asphalt pavements, which is shown in Figure 3a. The detail of PEH inside design is shown in Figure 3b. The components within the PEH include the packaging materials, the packaging materials, piezoelectric units and internal circuit board. The packaging material for the PEH was PA66-GF30, which was a type of nylon reinforced with 30% glass fiber. The PA66-GF30 was selected to serve as the upper and lower protective layers, in which the upper layer directly undertook the vehicle load, and the ground reaction force was supported by the lower layer. It was selected for the protective packaging of the PEH owing to its high toughness, load resistance, strength, and resistance to repeated shocks. A rubber gasket was employed between the upper and lower layers, which can prevent water leakage and reset the protective layers after loading. The piezoelectric ceramics are stacked to serve as the piezoelectric units between upper and lower layers, which are the core components of the PEHs. Within this internal circuit board, each power unit is connected to a full bridge rectifier and switched to an output bus after rectifying to reduce the adverse effects of uneven force [23], and these full bridge rectifiers are connected in parallel to output the generated voltage, as presented in Figure 3c.

As soon as vehicles pass cross on the PEH, the electrical energy could be produced. In terms of the contact patch of the tires and the thickness of asphalt pavements, the PEH was designed to have a square shape with a side length of 30 cm, and its thickness was 7 cm. The piezoelectric units are cylindrical structures with diameter of 2 cm and height of 2.25 cm.

### 3.3. Numerical Modeling of Piezoelectric Unit and Verification

**Figure 4:**
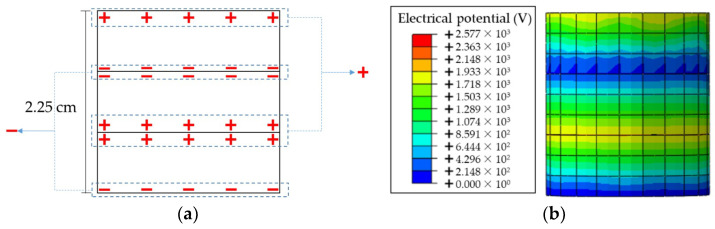
Piezoelectric unit used in this study: (**a**) Structure of piezoelectric unit; (**b**) Example of computational result.

Based on the research discovery from Cook-Chennault [29], the 3-3 working mode can achieve a higher energy conversion for PZT materials. According to the preliminary researches from Yang [30], the PZT-5H is a polycrystal made by lead titanate, lead zirconate and lead dioxide, which has a relative higher piezoelectric coefficients and compressive strength. Hence, in this study, three plates of PZT-5H with a thickness of 0.75 cm were electrically connected in parallel and the two adjacent contact surfaces have the same polarity, as shown in Figure 4a. Some parameters of the PZT-5H provided by the producer are listed in Table 1.

**Figure 5:**
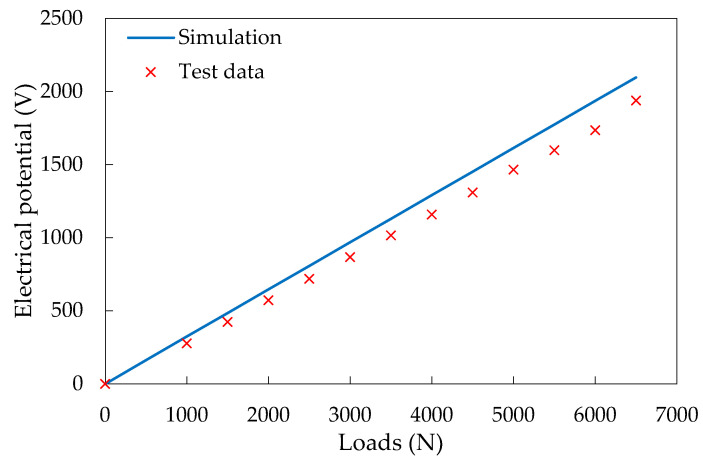
Validation of the piezoelectric unit simulation.

Before assembling the piezoelectric units into the PEH in the simulation, it is necessary to create the FE model of the piezoelectric unit first and verify the reliability of simulating its piezoelectric performance. To this end, the laboratory compressive loads were applied on the piezoelectric unit. The test was performed by the universal servo hydraulic test device (Cooper HYD25-II), which can randomly set the temperature and provide sinusoidal loads. During the tests, the sinusoidal loadings ranged from 1 to 6.5 kN with the interval of 0.5 kN were applied under loading frequency of 10 Hz and temperature of 20 °C [23]. Meanwhile, the FE model of the piezoelectric units was established in ABAQUS. The loading and boundaries conditions were defined as same as laboratory ones. The material parameters will be introduced in the next section. One example of the computational result is shown in Figure 4b. The distribution of the electrical potential is illustrated. In addition, Figure 5 compares the values of open-circuit current voltage from laboratory and the electrical potential from simulation.

The results show that the numerical results are consistent with the experimental results within this loading range. Therefore, the developed FE model of the piezoelectric unit can effectively predict its piezoelectric performance.

### 3.4. Development of PEH Finite Element Model

**Figure 6:**
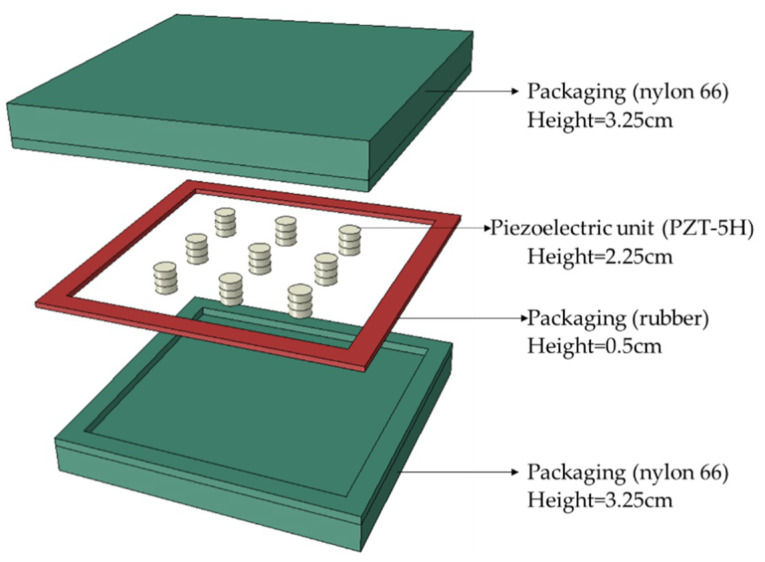
Overview of a PEH finite element model.

In the FE simulation of the PEH, the packaging and piezoelectric materials were modeled. To clearly exhibit the overview for a PEH FE model, Figure 6 shows the detailed constituents of the PEH model. In this model, tie bonding was assumed between packaging materials and between package and piezoelectric units, and therefore no slips and separations will occur.

**Figure 7:**
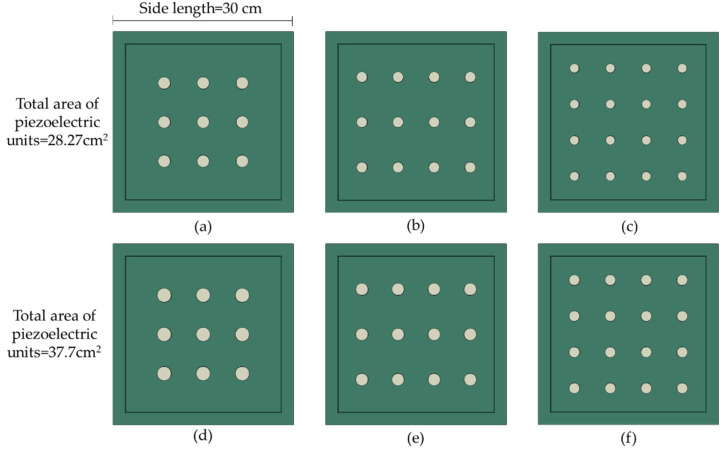
Piezoelectric unit distributions in PEH by: (**a**) 3 × 3 with diameter = 2 cm; (**b**) 3 × 4 with diameter = 1.73 cm; (**c**) 4 × 4 with diameter = 1.5 cm; (**d**) 3 × 3 with diameter = 2.3 cm; (**e**) 3 × 4 with diameter = 2 cm; (**f**) 4 × 4 with diameter = 1.73 cm.

To deeply investigate the efficiency of PEHs with different piezoelectric unit distributions, six different PEH FE models were developed as presented in Figure 7. To maximally utilize the space, the distributions of the piezoelectric units were designed in matrixes by 3 × 3, 3 × 4 and 4 × 4, respectively. According to previous researches [23,24,26], the cross section area of the piezoelectric units is related to the electrical potential; therefore, the total cross section area of the piezoelectric units was controlled in this study. For the distribution of 3 × 3 with diameter of 2 cm, 3 × 4 with diameter of 1.73 cm and 4 × 4 with diameter of 1.5 cm, the total cross section area of the units was 28.27 cm^2^. For the distribution of 3 × 3 with diameter of 2.3 cm, 3 × 4 with diameter of 2 cm and 4 × 4 with diameter of 1.73 cm, the total cross section area of the units was 37.7 cm^2^.

The elastic properties of PA66-GF30 and rubber were defined as typical values [31,32]. The piezoelectric properties for piezoelectric units (PZT-5H) were defined according to [27]. The parameters used in the simulation are listed in Table 2.

**Figure 8:**
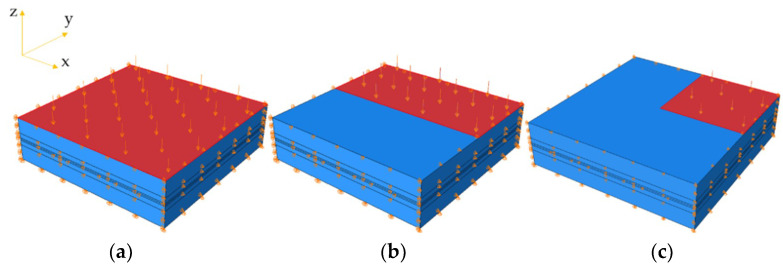
Loading modes on the PEH: (**a**) full loading; (**b**) half loading along y-direction; (**c**) quarter loading.

Based on a comprehensive mesh study, the element types for the packaging materials and piezoelectric units were C3D8 with size of 2 cm and C3D8E with size of 0.25 cm, respectively. To simulate the realistic loading conditions of the PEH in pavements, the bottom and sides of the PEH were restricted in vertical and horizontal directions, respectively. The uniform pressure was applied on the top of the PEH. To consider different traffic loading conditions from the moving vehicles, Figure 8 exhibits three modes of pressure loadings, including the full loading, half loading, and quarter loading.

For the distribution of 3 × 4, the half loading modes were, respectively, applied along x-direction (the loading area can fully cover 6 piezoelectric units) and along y-direction (the loading area can fully cover 4 piezoelectric units and partially cover another 4 piezoelectric units). The loading amplitude was 0.7 MPa.

## 4. Results and Discussion

### 4.1. Comparison of Piezoelectric Energy Production

According to [23], the electrical energy produced by the piezoelectric units can be calculated by

(3) $$E_{i}=\frac{1}{2}\frac{d_{z}^{2}\sigma_{z}^{2}A_{i}h}{\varepsilon }$$

where *d*_z_ is the piezoelectric coefficient in z-direction, C/N; $\sigma_{z}^{\;}$ is the stress in z-direction, Pa; ε is the dielectric constant, F/m; *A_i_* and *h* are the cross section area and height of *i*-th piezoelectric unit, m^2^ and m, respectively.

**Figure 9:**
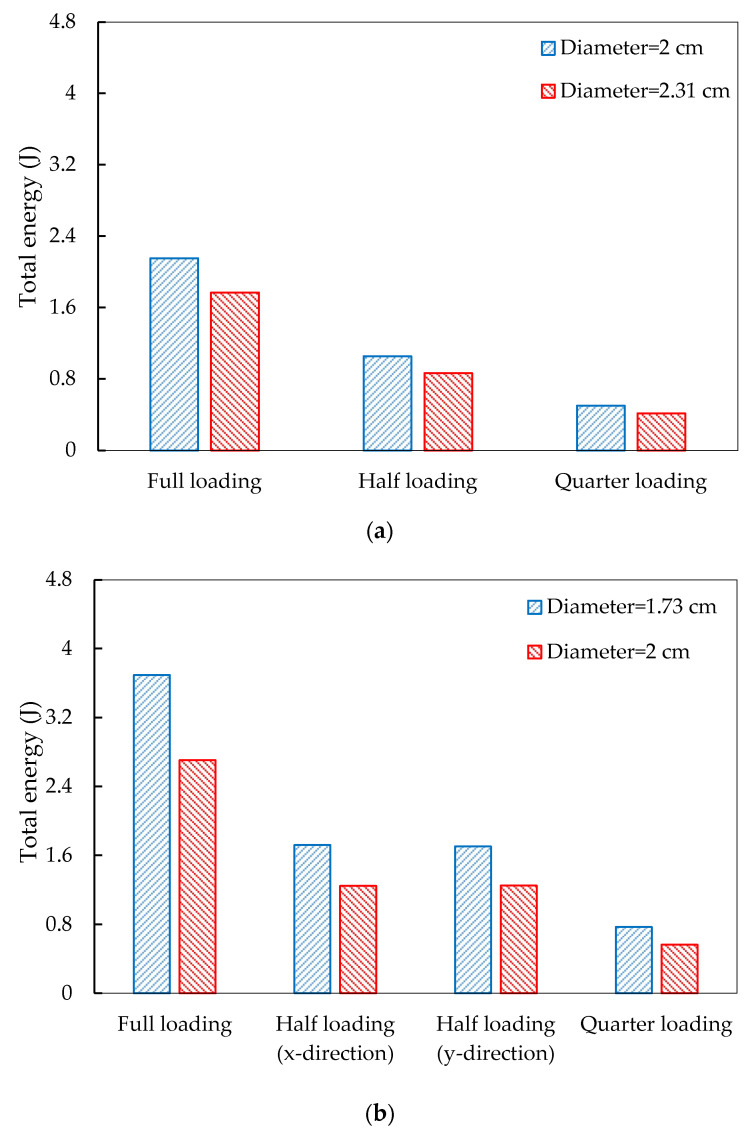

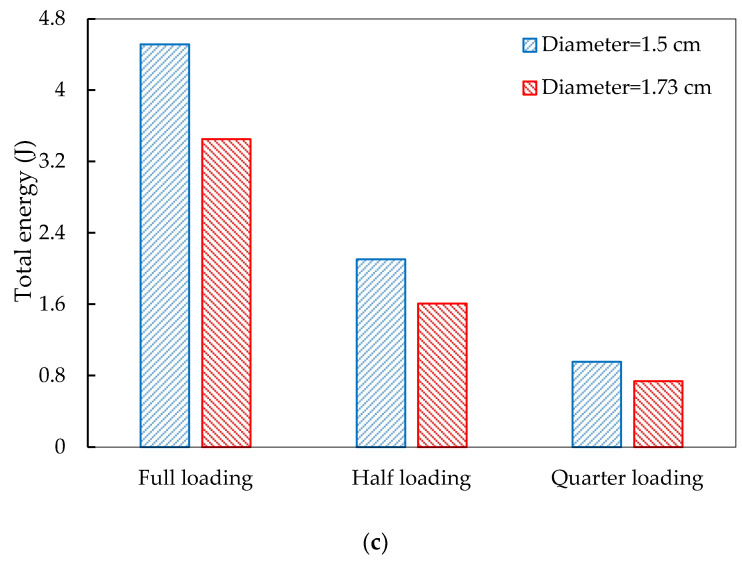
Total electrical energy production in the PEHs with different piezoelectric unit distribution: (**a**) 3 × 3; (**b**) 3 × 4; (**c**) 4 × 4.

To quantitatively exhibit the piezoelectric energy harvesting, Figure 9 exhibits the extracted total electrical energy production in the PEHs under the three loading modes.

It can be observed that higher electrical energy can be produced by piezoelectric units with smaller diameters. The total electrical energy production is linearly related to the loading conditions, i.e., the PEHs under half and quarter loading modes produced around half and quarter energy of that produced under full loading mode. For the piezoelectric units in 3 × 4 distribution, the total energy productions under half loading along x-direction and y-direction are equivalent. In addition, even the total cross section areas of the piezoelectric units are equivalent, the electrical energy production still shows large difference when the PEHs are under the same loading conditions. For instance, when the total cross section areas of the piezoelectric units are 28.27 cm^2^ (3 × 3 distribution with 2 cm diameter, 3 × 4 distribution with 1.73 cm diameter and 4 × 4 distribution with 1.5 cm diameter), however, they produced 2.15, 3.69, and 4.15 J electrical energy under full loading condition, respectively. This phenomenon can be explained by the stress distribution variations. According to Equation (3), the electrical energy is dependent on the stress response of the piezoelectric unit. Therefore, although the total cross section areas of the piezoelectric units in the three PEHs are equivalent, the stress conditions on units are different due to various distributions, and thus generate difference electrical energy.

### 4.2. Comparison of Electrical Potential

**Figure 10:**
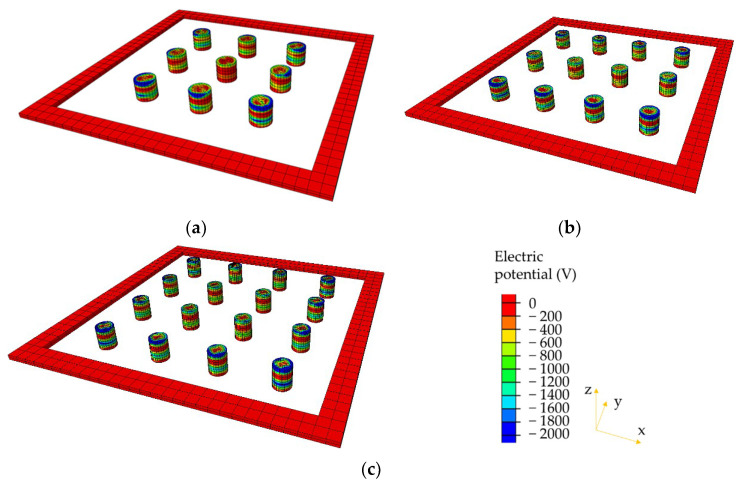
Electrical potential distributions in piezoelectric units under full loading condition: (**a**) 3 × 3 with diameter = 2 cm; (**b**) 3 × 4 with diameter = 1.73 cm; (**c**) 4 × 4 with diameter = 1.5 cm.

**Figure 11:**
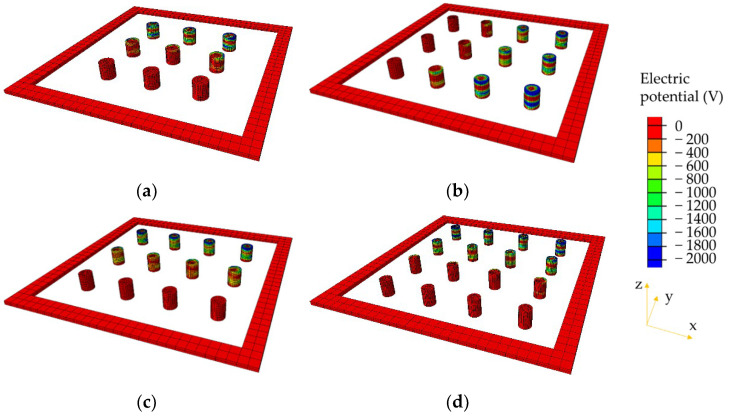
Electrical potential distributions in piezoelectric units under half loading condition: (**a**) 3 × 3 with diameter = 2 cm; (**b**) 3 × 4 with diameter = 1.73 cm, along x-direction; (**c**) 3 × 4 with diameter = 1.5 cm, along y-direction; (**d**) 4 × 4 with diameter = 1.5 cm.

**Figure 12:**
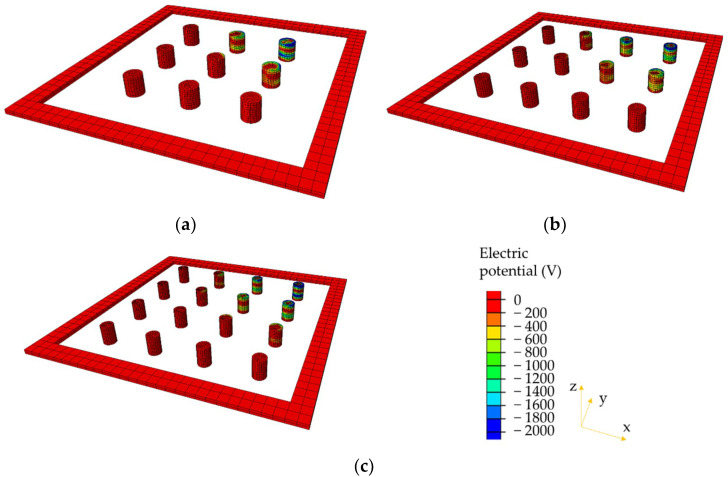
Electrical potential distributions in piezoelectric units under quarter loading condition: (**a**) 3 × 3 with diameter = 2 cm; (**b**) 3 × 4 with diameter = 1.73 cm; (**c**) 4 × 4 with diameter = 1.5 cm.

The electrical potential distributions within the piezoelectric units are, respectively, presented in Figure 10, Figure 11 and Figure 12. Only the models with smaller diameters (2, 1.73, and 1.5 cm) are compared here under the three loading modes. It can be observed that the electrical potential exhibits extremely non-uniform distribution in the PEHs. Piezoelectric units near the edges show relatively higher electrical potential than those in the centers of the PEHs. The aforementioned phenomenon can be explained by the stress concentration in the piezoelectric units near the edge. The stiffness of the piezoelectric units (PZT-5H) is much higher than that of the packaging and sealing materials (PA66-GF30 and rubber), and therefore, higher stress concentrations mostly exist in the units near the edge. Under the half and quarter loading conditions, even though the electrical potential in the piezoelectric units beyond the loading area is almost zero, very high electrical potential still appears near the edge of PEHs. These results indicate that the current PEH design will cause large difference in the electrical potential between different piezoelectric units, especially under the non-uniform loading conditions. The similar distributions of the electrical potential can be found in the other three models with larger diameters (2.31, 2, and 1.73 cm).

**Figure 13:**
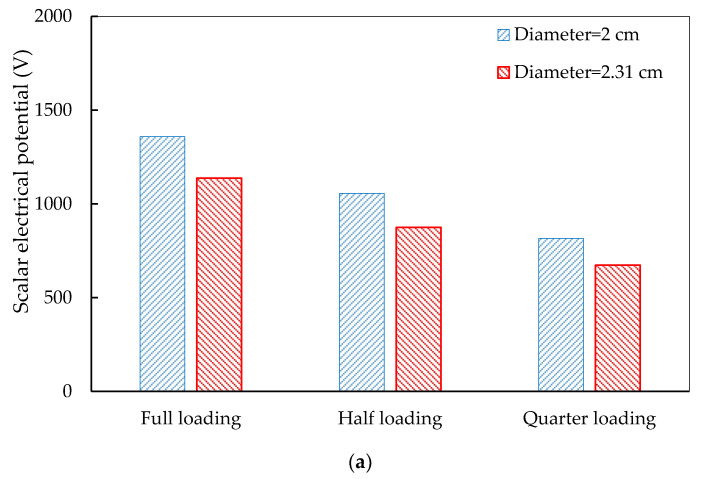

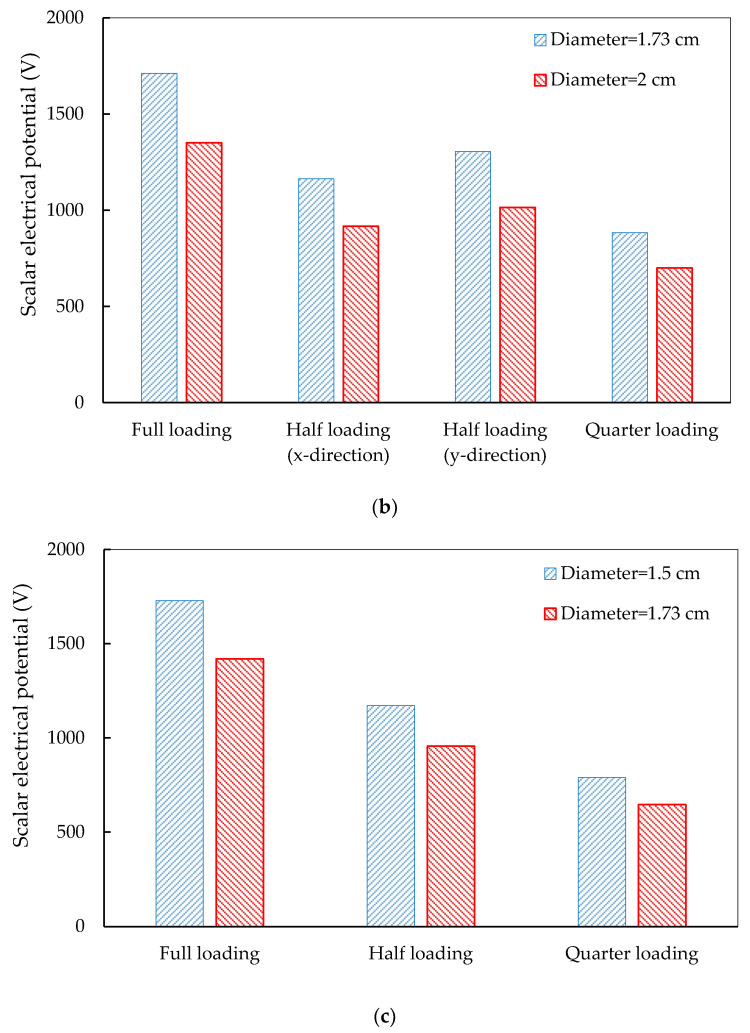
Electrical potential in the PEHs with different piezoelectric unit distributions: (**a**) 3 × 3; (**b**) 3 × 4; (**c**) 4 × 4.

As mentioned above, high difference in the electrical potential between piezoelectric units will reduce the efficiency of the energy harvesting. To evaluate the efficiency, average electrical potentials in the PEHs under the three loading modes are shown in Figure 13. It can be observed that the 4 × 4 distribution has the largest electrical potential, followed by 3 × 4 and 3 × 3 distributions. The results indicate that the electrical potential is highly related to the number of the piezoelectric units in PEHs.

In addition, the electrical potential difference between the piezoelectric units within one PEH is also an essential factor impacting the piezoelectric harvesting efficiency. In this study, the maximum and minimum electrical potentials are, respectively, produced by the piezoelectric units at the edge and center positions of the PEHs. The differences are listed in Table 3. Despite that the PEH with 4 × 4 distribution produced the highest average electrical potential in the piezoelectric units, largest potential differences can be observed in the electrical potentials under half and quarter loading modes. The PEHs with 3 × 4 piezoelectric unit distributions gain a better balance between the average electrical potential and the potential difference. In addition, the PEHs with 3 × 4 distribution under half loading mode along y-direction have higher electrical potential and smaller potential difference. This can be explained by the stress distributions. When the PEH bears the half loading along y-direction, four piezoelectric units near edge positions stand the stress concentration, which can generate higher electrical potential than that along x-direction. In addition, the difference between maximum and minimum electrical potential can be reduced by more piezoelectric units under the loading area. For the half loading along x-direction, six units were under the loading area; for the half loading along y-direction, eight units were under (or partially under) the loading area.

### 4.3. Comparison of Von Mises Stress

In engineer practice of PEH design, the mechanical responses of the piezoelectric units should also be considered to prevent or reduce the damages. To this end, the maximum von Mises stress in the piezoelectric units are derived and shown in Table 4 and Table 5. The two tables, respectively, include the piezoelectric units with smaller and larger diameters, i.e., the total cross section areas of the piezoelectric units, respectively, are 28.37 cm^2^ for Table 4 and 37.7 cm^2^ for Table 5. The maximum von Mises stress always exists near the edge of the PEHs. From these results, it can be observed that the highest von Mises stress occurs in the PEH with piezoelectric units in 3 × 3 distribution, which indicates that less piezoelectric units would induce higher von Mises stress. However, the piezoelectric units with 3 × 4 distributions have slightly lower von Mises values than that with 4 × 4 distributions. This can be explained by the detailed spatial arrangement of the piezoelectric units in the two PEHs. As one can see in Figure 7, the piezoelectric units with 4 × 4 distribution were closer to the edge than that with 3 × 4 distribution, and hence higher stress concentration exists in the 4 × 4 distribution. For the same piezoelectric unit distribution, the larger cross section area can effectively reduce the von Mises stress.

### 4.4. Evaluation of the Piezoelectric Effect

**Figure 14:**
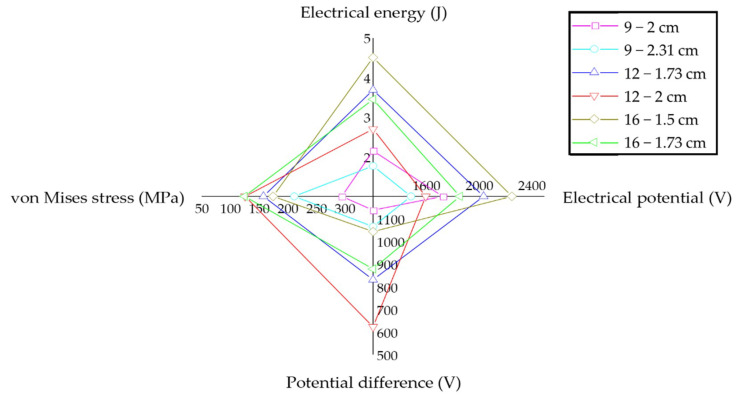
Radar chart of the output value of PEHs under full loading model.

To evaluate the piezoelectric effects of different PEHs, a radar chart was provided in Figure 14, in which the output electrical energy, electrical potential, potential difference, and von Mises stress of the PEHs under the full loading mode were exhibited.

According to previous researches [23,24], higher electrical energy and potential are required for piezoelectric energy harvesting. In addition, larger von Mises stress and potential difference can, respectively, increase the damage behavior of the piezoelectric units and decrease the energy harvesting efficiency. It can be observed that the PEHs with more piezoelectric units can produce higher electrical energy and potential, and meanwhile reduce the von Mises stress concentration and potential difference. However, the PEHs with 4 × 4 distributions experience higher von Mises stress and potential difference than that with 3 × 4 distributions, which could be caused by the spatial locations of the piezoelectric units. Amongst the six PEHs, the 3 × 4 distribution with smaller cross section of the piezoelectric units achieves a better balance between electrical energy harvesting and stress concentration.

## 5. Conclusions and Outlook

In this study, different PEHs with different piezoelectric unit distributions were modeled using FE method to evaluate their efficiency of energy harvesting. The PEH model in the simulation includes piezoelectric units and packaging materials. In total, three different piezoelectric unit distributions (3 × 3, 3 × 4 and 4 × 4) were developed. In addition, different cross section areas (28.27 and 37.7 cm^2^) of the piezoelectric units were considered. To simulate the loading and boundary condition of PEH in asphalt pavements, three loading modes were applied on the PEH models (full, half, and quarter loading modes).

To sum up, the PEH with more piezoelectric units can increase the non-uniformity of the stress distribution, and produce more electrical power. Furthermore, higher electrical potential can be produced by piezoelectric units with smaller cross section area. The PEH with piezoelectric units in 4 × 4 distribution can generate more power from the traffic loads. However, remarkable electrical potential difference can be observed in the PEH with 4 × 4 distribution especially under non-uniform loading conditions. The highest electrical potential occurs near the edge of PEHs whilst the piezoelectric units in the center position produced the lowest electrical potential. In addition, larger cross section area could significantly reduce the electrical potential in the piezoelectric units. The stress results indicate that less piezoelectric units normally induce higher von Mises stress in PEHs. For the same distribution, the von Mises stress can be reduced by increasing the cross section area of the piezoelectric units.

Based on the aforementioned conclusions, for the PEH design in the future, some recommendations are proposed. When the total cross section areas of the piezoelectric units are the same, in order to produce higher energy, more piezoelectric units are suggested to be used. In addition, the diameter of the piezoelectric units near the edge could be larger than those at the center of the PEH, which would not only reduce the difference in the electrical potential between piezoelectric units at edge and center positions, but also effectively prevent or reduce the high stress concentration at edge positions.

## Figures and Tables

**Table 1:** Basic parameters for PZT-5H.

Parameters	Value
Density (kg/m^3^)	7500
Poisson’s ratio	0.3
Electromechanical coupling factor k_p_	0.65
Mechanical quality factor Q_m_	70
Curie temperature Tc (°C)	200

**Table 2:** Model parameters.

	PZT-5H	PA66-GF30	Rubber
Elastic constants (Pa)	C_11_ = 12.6 × 10^10^ C_12_ = 5.50 × 10^10^ C_13_ = 5.30 × 10^10^ C_33_ = 11.7 × 10^10^ C_44_ = 3.53 × 10^10^	–	–
Piezoelectric constants (C/m^2^)	e_31_ = −6.5 e_33_ = 23.3 e_15_ = 17.0	–	–
Dielectric constants (C/(V·m))	ε_11_ = 1.511 × 10^-8^ ε_33_ = 1.301 × 10^-8^	–	–
Elastic modulus (Pa)	–	5.9 × 10^9^	8 × 10^6^
Poisson’s ratio	–	0.35	0.47

**Table 3:** Electrical potential difference in PEHs.

Piezoelectric Units	Loading Modes	Maximum Electrical Potential (V)	Minimum Electrical Potential (V)	Difference (V)
3 × 3 Diameter = 2 cm	Full loading	1728	589	1139
	Half loading	1777	296	1481
	Quarter loading	1821	144	1677
3 × 3 Diameter = 2.31 cm	Full loading	1487	421	1066
	Half loading	1528	206	1322
	Quarter loading	1562	99	1463
3 × 4 Diameter = 1.73 cm	Full loading	2038	1207	831
	Half loading (x-direction)	2108	180	1928
	Half loading (y-direction)	2079	598	1481
	Quarter loading	2130	86	2044
3 × 4 Diameter = 2 cm	Full loading	1599	976	623
	Half loading (x-direction)	1643	150	1493
	Half loading (y-direction)	1628	488	1140
	Quarter loading	1666	73	1593
4 × 4 Diameter = 1.5 cm	Full loading	2253	1209	1044
	Half loading	2308	188	2120
	Quarter loading	2346	20	2326
4 × 4 Diameter = 1.71 cm	Full loading	1859	980	879
	Half loading	1904	139	1765
	Quarter loading	1934	11	1923

**Table 4:** Maximum von Mises stress in PEHs. (total cross section area = 28.37 cm^2^).

Distribution	Diameter (cm)	Von Mises Stress (MPa)		
		Full Loading	Half Loading	Quarter Loading
3 × 3	2	295	288	260
3 × 4	1.73	158	136 (x-direction) 133 (y-direction)	134
4 × 4	1.5	175	173	173

**Table 5:** Maximum von Mises stress in PEHs. (total cross section area = 37.7 cm^2^).

Distribution	Diameter (cm)	Von Mises Stress (MPa)		
		Full Loading	Half Loading	Quarter Loading
3 × 3	2.31	212	211	150
3 × 4	2	126	137 (x-direction) 124 (y-direction)	113
4 × 4	1.73	125	117	119

## Data Availability

The data presented in this study are available on request from the corresponding author. The data are not publicly available due to project requirement.
